# A Sandwich Electrochemical Immunosensor Using Magnetic DNA Nanoprobes for Carcinoembryonic Antigen

**DOI:** 10.3390/ijms12117410

**Published:** 2011-10-28

**Authors:** Ning Gan, Liyong Jia, Lei Zheng

**Affiliations:** 1 The State Key Laboratory Base of Novel Functional Materials and Preparation Science, Faculty of Material Science and Chemical Engineering, Ningbo University, Ningbo 315211, Zhejiang, China; E-Mail: Jialiyong@163.com; 2 Clinical Laboratory Center, Nanfang Hospital, Southern Medical University, Guangzhou 510515, Guangdong, China

**Keywords:** chitosan/DNA/Fe_3_O_4_/ZrO_2_, magnetic nanoparticle-based electrochemical immunoassay, carcinoembryonic antigen, sandwich electrochemical immunosensor

## Abstract

A novel magnetic nanoparticle-based electrochemical immunoassay of carcinoembryonic antigen (CEA) was designed as a model using CEA antibody-functionalized magnetic beads [DNA/Fe_3_O_4_/ZrO_2_; Fe_3_O_4_ (core)/ZrO_2_ (shell) nano particles (ZMPs)] as immunosensing probes. To design the immunoassay, the CEA antibody and *O*-phenylenediamine (OPD) were initially immobilized on a chitosan/nano gold composite membrane on a glassy carbon electrode (GCE/CS-nano Au), which was used for CEA recognition. Then, horseradish peroxidase (HRP)-labeled anti-CEA antibodies (HRP-CEA Ab_2_) were bound to the surface of the synthesized magnetic ZMP nanoparticles as signal tag. Thus, the sandwich-type immune complex could be formed between secondary antibody (Ab_2_) modified DNA/ZMPs nanochains tagged by HRP and GCE/CS-nano Au. Unlike conventional nanoparticle-based electrochemical immunoassays, the recognition elements of this immunoassay included both electron mediators and enzyme labels, which obviously simplifies the electrochemical measurement process. The sandwich-type immunoassay format was used for online formation of the immunocomplex of CEA captured in the detection cell with an external magnet. The electrochemical signals derived from HRP during the reduction of H_2_O_2_ with OPD as electron mediator were measured. The method displayed a high sensitivity for CEA detection in the range of 0.008–200 ng/mL, with a detection limit of 5 pg/mL (estimated at a signal-to-noise ratio of 3). The precision, reproducibility, and stability of the immunoassay were good. The use of the assay was evaluated with clinical serum samples, and the results were in excellent accordance with those obtained using the standard enzyme-linked immunosorbent assay (ELISA) method. Thus, the magnetic nanoparticle-based assay format is a promising approach for clinical applications, and it could be further developed for the detection of other biomarkers in cancer diagnosis.

## 1. Introduction

Carcinoembryonic antigen (CEA) is a protein found in many types of cells, but it is specifically associated with tumors and the developing fetus. The normal range of the CEA level in blood is 2~4 ng/mL in adult non-smokers [1–3]. CEA is very important in tumor screening and diagnosis, and the increasing demand for its early and ultrasensitive detection has driven the development of novel detection technologies. Among the various analytical techniques available, electrochemical biosensing, particularly electrochemical immunosensor (ECI), has gained considerable interest and has been used extensively to determine the serum CEA level because of its intrinsic advantages such as its portability, low cost, high sensitivity, and high specific molecular recognition of immunoreactions [2–5]. The sandwich-type ECI for CEA has recently been developed [6]. In the detection scheme, the primary antibody and enzyme (such as horseradish peroxidase [HRP])-labeled antibody recognize the antigen together to produce a “sandwich”-type immune complex. However, the signal generated from the enzyme-labeled antibody can catalyze a specific substrate, whose signal increases with an increase in the antigen concentration. Thus, the concentration of antigen can be determined by this method [7–11]. The key incentive for constructing this kind of sensor was to easily synthesize probes with a high concentration of antibody and enzyme for signal amplification.

Antibody immobilization is vital for the successful development of an ECI. Because of their excellent biocompatibility and stable electrochemical properties, many kinds of electrically conducting nanoparticles (NPs), such as nano gold (Au NPs), platinum, and silver, have attracted increasing attention for antibody immobilization and the subsequent development of an ECI. In recent years, For example, nano-sized gold particles possess some outstanding properties of large surface-to-volume ratio, high electron communication, and excellent biocompatibility, which are well documented to be suitably used as the enzyme-loading materials in various biosensor designs [11–14]. Au NPs-modified electrode surfaces can generally be prepared in two ways: electrostatic adsorption and covalent bonding [14]. The former method is simple, but the formed Au NPs layer is often unstable. In the current work, we firstly introduced nano gold crystals into the chitosan (CS) matrix, then casted it onto the GCE electrode so as to provide larger surface area for anchoring anti CEA to prepare CEA probes, which can result in a stable immune sensing interface for the direct and sensitive detection of CEA [15–28].

In the present study, an ECI was prepared by using a CS membrane to electrostatically adsorb Au NPs onto a glassy carbon electrode (GCE). The process involved two steps: first, nano-Au immobilized onto the GCE/CS surface, which comprised the CS/nano-Au monolayer. Then, the first CEA antibody (anti-CEA) was adsorbed onto the surface of the nano-Au layer, and bovine serum albumin (BSA) was used to block any remaining active sites on the nano-Au monolayer. Recently, zirconia nanoparticles (ZrO_2_ NPs) were used as selective sorbents to capture the bimolecular with phosphate group, such as DNA, and with carboxylic group, such as enzymes [15–16,29,30]. Further, owing to the selective affinity between Zr and carboxyl groups, nano ZrO_2_ can also be conjugated to monoclonal antibodies and enzymes, which are then used as labeling agents for antigen detection. Because of their enriched enzyme capacity, the probes can amplify the catalysis of a particular electrochemical reaction to produce current signals. DNA has good conductivity and bio-compatibility, and one DNA chain can immobilize a substantial amount of nano ZrO_2_, in the preparation of polymer chain probes. However, if DNA is directly used to fix ZrO_2_ in probe preparation, it is very difficult to separate the probes from the free antibodies that coexist in the suspension. In recent years, because of the widespread applications of magnetic nanoparticles (MNPs) in biomedical, biotechnology areas [17], much attention has been paid to the preparation of different kinds of MNPs. Among them, nano ferromagnetic probes composed of nano ferromagnetic oxides (such as Fe_3_O_4_) have been developed quickly. This method is convenient, simple and rapid [18]. Our group has synthesized Fe_3_O_4_ (nuclear)/ZrO_2_ (shell) magnetic nano spheres (abbreviated as ZMPs) that are suitable for immobilizing antibodies [18]. DNA, in the form of DNA-linked long chain molecules, contains several phosphate groups, on which magnetic beads of HRP-labeled CEA antibody can be fixed.

In this study, we used DNA as a template to prepare magnetic “cross-linked” nanochain probes (DNA/(ZMPs-HRP-CEA Ab_2_)*_n_*) to produce a novel sandwich ECI for CEA. After specifically interacting with CEA in the sample solution, the ECI was incubated with DNA-labeled ZMPs/HRP-CEA Ab_2_ antibody to allow the formation of a sandwich complex with the capture antibody CEA DNA/(ZMPs-HRP-CEA Ab_2_)*_n_*. Then, the HRP enzyme enriched at the electrode surface catalyzed the OPD oxidation by hydrogen peroxide, which produced an amplified reduction peak through an electron-transfer reaction. The response current was directly related to the concentration of the analyte, *i.e*., human CEA.

## 2. Results and Discussion

### 2.1. Characterization of Different NP Complexes

**Figure 1:**
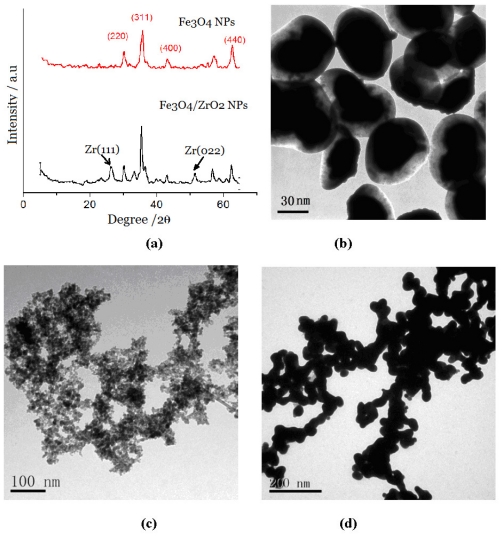
(**a**) XRD of ZMP particles and TEM images of (**b**) ZMP particles; (**c**) ZMP-HRP-CEA Ab_2_; and (**d**) magnetic cross-liked nanochains (DNA/ (ZMPs-HRP-CEA Ab_2_)*_n_*).

The ZMP nanocomposites were characterized by X-ray diffraction (XRD) and transmission electron microscopy (TEM). The XRD graph (Figure 1a) showed the characteristic peaks of both Fe_3_O_4_ (311, 2θ = 37.5°) and ZrO_2_ (111, 2θ = 27.5°; 022, 2θ = 52°) crystals, which indicated that the ZrO_2_ wrapped in a layer of Fe_3_O_4_ is in the crystalline state. X-ray fluorescence spectrum (XRF) analysis of the ZMPs showed Zr-K_β_(17.8 keV), Zr-K_α_(15.8 keV), Zr-L_β_ (2.1 keV), Zr-L_α_ (2.0 keV), Fe-K_β_ (7.1 KeV), and Fe-K_α_ (6.4 keV) peaks (data not shown), which indicated that elemental Fe and Zr exist in these magnetic particles. TEM (Figure 1b) showed that the ZMP structure was of the core (black)-shell (white) type, with Fe_3_O_4_ being the nucleus and ZrO_2_ being the shell. When the ZMP surface was coated with a layer of antibody, these two components unite (Figure 1c).

**Figure 2:**
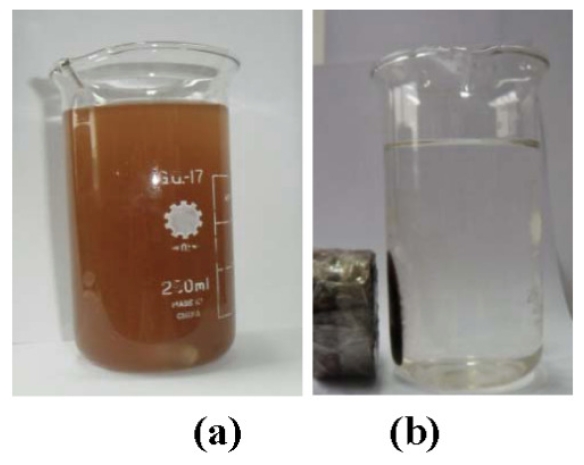
Nanoprobes absorbed before and under the external magnetic field.

As shown in (Figure 1d), the probe showed the appearance of a cross-linked “bead chain” because the DNA molecules that absorbed the ZMP were cross-linked with each other in solution. The ZMPs then wrapped around the DNA, forming cross-linked bead chains. XRF showed that the chain contained Zr-k_α_ (2.1 keV), Fe-k_α_ (6.4 keV), P-k_α_ peak (1.13 keV), and S-k peaks (2.3 keV) (data not shown). Because DNA is rich in phosphoric acid, the presence of P-k_α_ peak groups on the ZMP surface further confirmed that the ZMPs coated the DNA. We examined the ultraviolet spectrum after the reaction between the ZMPs and DNA and found that the function absorption peaks of DNA redshifted from 520 nm to 524 nm, implying that the DNA and ZMPs had interacted and this led to the function absorption redshift. Figure 2 shows that the DNA/(ZMPs-HRP-CEA Ab_2_)*_n_* magnetic probe has good superparamagnetism under the reaction of the 0.3 mTde external magnetic field.

### 2.2. Electrochemical Behaviors of the ECI

**Figure 3:**
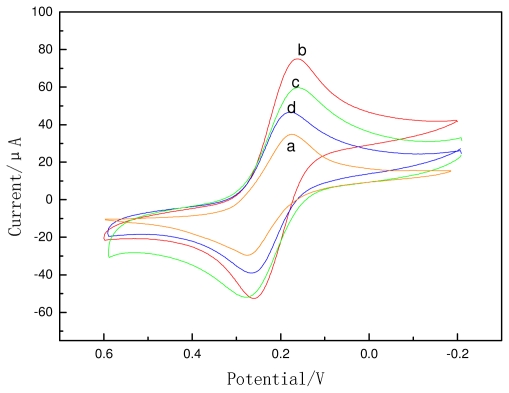
Cyclic voltammograms of GCE modified with (**a**) CS, (**b**) CS/nano-Au, (**c**) CS/nano-Au /Ab_1_, and (**d**) CS/nano-Au/Ab_1_/BSA in PBS (pH 7.0) containing 1 mmol/L Fe(CN)_6_^3–/4–^.

Figure 3 shows the electrochemical characterization of differently modified electrodes in 1 mM Fe(CN)_6_ ^3–/4–^. GCE modified with CS showed a redox peak with a large peak-to-peak separation (curve a). When nano-Au was modified on the electrode, the anodic and cathodic current peaks increased, and the peak-to-peak separation reduced (curve b); an explanation for this could be that nano-Au accelerates electron transfer. When the Ab_1_ adsorbed onto nano-Au (curve c), the current decreased, because the antibody may hinder electron transfer. When the electrode was incubated with BSA (curve d), the peaks declined further.

**Figure 4:**
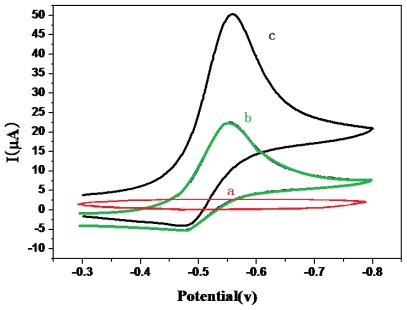
Cyclic voltammograms of (**a**) GCE/Ab_1_ in PBS (pH 7.0) and (**b**) GCE/Ab_1_/CEA and (**c**) GCE/Ab_1_/CEA/(DNA/(ZMPs-HRP-CEA Ab_2_)*_n_*) in PBS containing 5 mmol/L H_2_O_2_.

**Figure 5:**
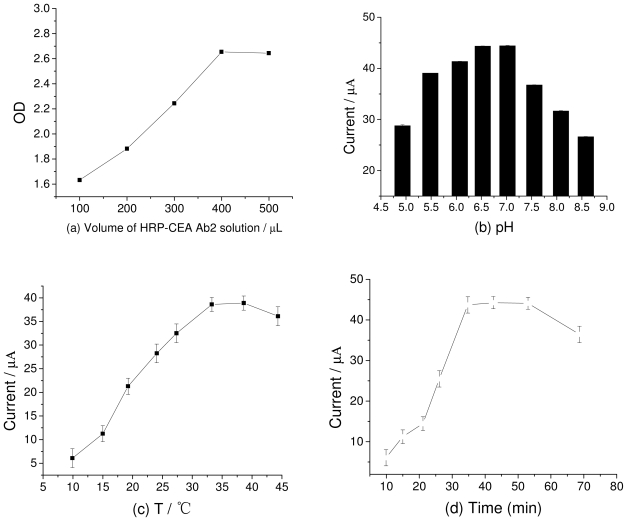
(**a)** OD values with different amounts of HRP-CEA Ab_2_; (**b**) Influence of pH; (**c**) Incubation temperature and (**d**) Incubation time taken for the immunosensor to respond.

As shown in Figure 4, the GCE/CS-Au NPs/Ab_1_ electrode exhibited no redox peak in the blank (PBS) between −0.3 V and 0.8 V. When 5 mmol/L H_2_O_2_ was added into the solution, the modified electrode (Figure 5b) showed a pair of redox peak (100 mV/s) between −0.48 V and −0.55 V. When the immunosensor was used to test CEA and the sandwich immune complex was formed (Figure 4c), the reduction current of the OPD oxidization complex increased obviously, and the oxidation current reduced. This is because the HRP enzyme on the surface of the probe catalyzes the reaction between H_2_O_2_ and OPD and increases the detection signal.

### 2.3. Optimization of Experimental Conditions

The concentration of HRP immobilized on the ZMPs is critical for the performance of the immunosensor because HRP catalyzes the reduction of OPD by H_2_O_2_. To obtain the optimum amount of HRP-labeled and tagged antibody on ZMPs, we compared the OD values of HRP-CEA Ab_2_ labeled on ZMPs by using the enzyme-linked immunosorbent assay (ELISA) at 450 nm. We separately added 100, 200, 300, 400, and 500 μL of 12 μg/L HRP-CEA Ab_2_ into 5 mL of 2 mg/mL ZMP NPs and then added 1 mmol/L H_2_O_2_ for detection. As seen from (Figure 5a), the OD values of HRP-CEA Ab_2_ increased as its concentration increased. When 400 μL HRP-CEA Ab_2_ (4.8 ng) was added, the OD values stabilized, indicating that the adsorption of HRP in ZMPs reaches saturation at 400 μL.

The effect of the electrolyte pH in the ECI is examined from two aspects: First, it directly influences the activity of HRP. Second, it affects the peak potential of the electrode reaction. (Figure 5b) shows the magnitude of the catalytic current from modified electrodes with electrolyte of different pH values. When the pH value was 7.0, the catalytic current was the maximum. This indicates that when the pH is 7.0, the HRP activity in the modified electrode membrane is the highest. These findings are consistent with the original nature of HRP [17]. Thus, the progress of the enrichment of ZMPs with HRP does not change the pH value at which the maximum catalytic reactions of HRP occur. On mapping the reduction and oxidation peak potentials against the pH values, we obtained two lines between pH 4 and 8. Their slopes were −59.87 and −51.72 mV/pH unit. The peak potential increased with pH, indicating that protons participate in the electrode reaction of OPD oxidation. The ratio of the participating protons and the electrons transferred in the electrode reaction was 1:1. Since the process of the electrode response for OPD was a double-electron process, the number of protons participating in the electrode reaction is 2 [18], which is the same as the number of electrons gained/lost in the redoxidation of OPD.

The effect of the incubation temperature (Figure 5c) on the catalytic current was also studied. The immunosensor was found to possess a good current response signal at room temperature. Thus, all the experiments were conducted at room temperature. The incubation time of CEA Ab_2_ with functionalized DNA/ZMPs-Au was also optimized (Figure 5d), and the results showed that the maximum time required was 30 min. Thus, the optimum experimental conditions for detection were found to be pH 7.0, room temperature, and an incubation time of 30 min.

### 2.4. Detection of CEA

**Figure 6:**
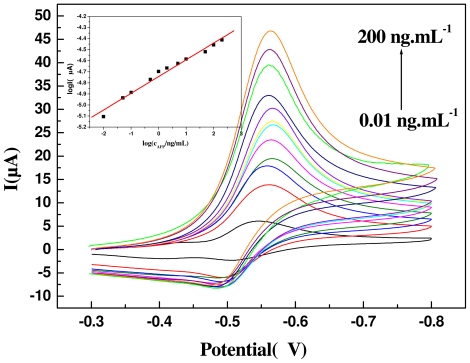
Differential cyclic voltammetric curves. Insert: The calibration curve of the ECI with different concentrations of CEA under optimal experimental conditions.

As shown in Figure 6, the response current increased with the increase in the CEA concentration. When the concentration ranged from 0.008 to 200 ng/mL, the linear correlation coefficient was 0.9943. The detection limit (DL) was 5 pg/mL (3δ). The average level of CEA in healthy humans is 20 ng/mL, so our ECI can easily be used for CEA detection in lab samples without any need for dilution.

Next, we compared our sensor with other sandwich-type sensors. As shown in Table 1, most sandwich immunosensors have a low DL and high detection sensitivity, and their DL is 100 times lower than that of traditional ELISAs.

### 2.5. Precision, Reproducibility, and Stability

The immunosensors prepared at different times and in different batches were used to test 15 and 25 ng/mL CEA four times. The coefficient of variation obtained was 2.3% and 2.2%, respectively, indicating the good precision of the immunosensors. The proposed sensors were stored in PBS (pH 6.5; 4 °C) for 45 days, and they did not show any obvious changes (signal changes <5%), which shows that these sensors have good storage stability. The electrode surface can be updated by placing the electrode under the traction of a magnetic field (0.3 mT at the bottom of the electrolytic cell, with the magnetic field lines oriented perpendicular to the direction of the surface of the electrode in solution) and then mixing the solution in order to free the magnetic nanoprobes from the electrode surface. Updated electrodes can be used again for sandwich detection. Such electrodes were used to test 15 and 100 ng/mL CEA samples, and the measured values were 15.7 and 99.3 ng/mL, respectively, with relative standard deviation values (*n* = 3) of 3.1% and 3.2%, respectively. These findings suggest that the ECIs have good preparation repeatability.

We studied the effects of major interfering agents in blood serum on the immunosensor. When the concentration of CEA was 5 ng/mL, the difference in the sensor signal deviated only 5% from the normal with the following interfering agents: 10 times the normal level of CEA and hepatitis B virus; 200 times the normal level of BSA, glucose, and uric acid; and 800 times the normal levels of Na^+^, Fe^2+^, Fe^3+^, Zn^2+^, and Ca^2+^. This suggests that the sensor effectively resists disturbances caused by the main interfering agents in human serum.

### 2.6. Application of the Immunosensor for Detecting CEA in Human Serum

As shown in Table 2, the level of CEA in human serum was determined using our ECI. The results were consistent with those obtained using ELISA, and the recoveries were between 95% and 107%, indicating that our method is suitable for detecting CEA in serum. The DL of the ECI can reach 5 pg/mL, while that of ELISA can only reach a maximum of 0.1 ng/mL. Thus, our method is 100-fold more sensitive than ELISA. Therefore, it is fit for detecting minor changes in the CEA concentration in serum, which is in turn useful for the early diagnosis of cancer.

## 3. Experimental Section

### 3.1. Reagents and Chemicals

OPD and H_2_O_2_ were purchased from Shanghai Crystal Pure Reagent Co. Ltd in China. We made the Fe_3_O_4_/ZrO_2_ magnetic particles by ourselves. CEA monoclonal antibody solution (Ab_1_) and CEA ELISA kits were obtained from Biocell (Zhengzhou, China); the latter contain standard CEA solution and HRP-labeled CEA monoclonal antibody (HRP-Ab_2_). 1 mg/mL Nano gold colloid (5 nm) Calf thymus DNA and BSA were purchased from Aldrich (Sigma Co. Ltd, USA). All other reagents were of analytical grade. Double-distilled water was used for all experiments.

### 3.2. Apparatus

Cyclic voltammetric measurements were performed on a CHI 660a electrochemistry workstation (Shanghai CH Instruments, China) with a three-electrode system composed of a platinum wire auxiliary electrode, a saturated calomel reference electrode (SCE), and a bare or modified GCE/CS/nano-Au electrode as the working electrode. Scanning electron micrographs were taken with a scanning electron microscope (SEM; S-4800; Hitachi, Tokyo, Japan). Further, we used an S2 RANGER X-ray fluorescence spectrometer (Bruker, Germany), ST-360 microplate reader (Shanghai Science Biotechnology), NdFeB magnet (Hangzhou Magnet Equipment Ltd., China), and an ultra-pure water meter (Millipore, USA).

### 3.3. Synthesis of Magnetic Cross-Liked Nanochain Probes

First, colloidal nano ZrO_2_ and nano Fe_3_O_4_ particles were synthesized as reported previously [29,30]. Next, 1 g nano Fe_3_O_4_ particles were added into 100 mL of 10 mg/mL colloidal nano ZrO_2_, and the mixture was stirred for 6 h at room temperature. The resulting particles were separated by applying an external magnetic field. The maximum size of the ZMPs was 18.6 nm (93.2% of the total number of particles), and the minimum was 45.83 nm (6.8% of the total number of particles). Second, HRP-Ab_2_ was added to 5 mL of 2 mg/mL ZMP solution, the mixture was stirred constantly for 12 h, and the unabsorbed Ab_2_ was eliminated by magnetic separation. The final mixture was washed repeatedly. Subsequently, HRP was used to tagged unreacted and nonspecific sites on the ZMPs. Magnetic cross-linked nanochains (DNA/(ZMPs-HRP-CEA Ab2)*_n_*) were obtained from a mixture of 10 mg/mL calf thymus DNA and ZMPs-HRP-CEA Ab_2_ that was gently stirred in a magnetic field for 6 h and then separated magnetically. The obtained probes were resuspended in PBS of pH 7.0 containing 0.2% BSA. The formation of this probe was monitored by TEM.

### 3.4. Designing of the Immunosensor Immunoassay Procedure for CEA Detection

**Scheme 1:**
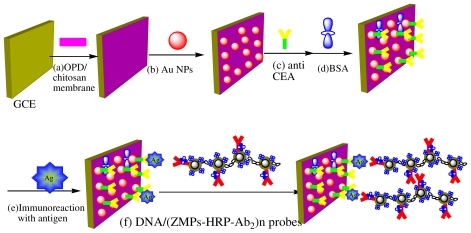
Schematic processes of the immunosensor fabrication process and detection principle. (**a**) Dropping of CS membrane; (**b**) electrochemical deposition of HAuCl_4_; (**c**,**d**) immobilization of Ab_1_ and blocking with BSA; (**e**) immunoreaction of CEA, (**f**) incubation in solution containing DNA/(ZMPs-HRP-CEA Ab_2_)*_n_*.

First, 10 μL of 0.5% CS ethanol was deposited onto the polished GCE surface. The GCE/CS obtained when the ethanol was volatilized completely was washed with double-distilled water. Second, 50 μL 1 mg/mL nano-Au (5 nm) was electrochemically deposited onto the CS membrane surface; Finally, GCE/CS/nano-Au was obtained, and the electrode was scanned 10 times with the potential increasing from −0.8 to 0.8 V in 0.1 M NaOH solution at 100 mV/s. Then, this electrode was soaked in 0.1 M PBS (pH 7.0) containing 1 mg/mL Ab1 for 12 h, and the GCE/CS/nano-Au/Ab1 (hereafter referred to as GCE/Ab1) immunoelectrode was obtained. The electrode was incubated with 3 mg/mL BSA for 30 min to block the unreacted and nonspecific sites (Scheme 1).

### 3.5. Immunoassay Procedure for CEA Detection

Scheme 1 shows the detection principle of the immunosensor. The procedure is based on the typical procedure used in sandwich-type immunoreactions. When the immunoreaction between GCE/Ab_1_ and the CEA antigen was completed, DNA/(ZMPs-HRP-Ab_2_)_n_ probes were captured through the “sandwich” mode to form sandwich-type immune complexes (Ab_1_/CEA/(DNA/(ZMPs-HRP-Ab_2_)_n_). The response current of this immunosensor was found to be positively correlated with CEA in a certain concentration range. Therefore, it could be used for the quantification of CEA, because the HRP on the surface of the immune complex can catalyze the reaction between H_2_O_2_ and OPD.

## 4. Conclusions

In summary, we developed a rapid and sensitive immunoassay using a new kind of sandwich amperometric immunosensor combined with the usage of magnetic DNA-tagged nanoprobes. To enhance detection sensitivity, we pursued a multienzyme labeling strategy instead of a single-enzyme label during the immunoassay. The achieved amplification of signal was ascribed to a large amount of HRP and HRP labeled CEA antibody loaded on the ZMPs nanocarrier. This immunoassay also has the following advantages: (1) By using Fe_3_O_4_/ZrO_2_ magnetic beads to absorb the enzyme-linked antibody, the unlabeled antibody can be quickly and easily separated by exposure to the external magnetic field. This simplifies the process of probe preparation. Further, owing to the large specific surface area of Fe_3_O_4_/ZrO_2_, the density of HRP on the probes is rather high. Thus, the sensitivity of the immunosensor is enhanced; (2) With the specific adsorb-ability of DNA and ZrO_2_, one DNA chain can bridge more than one ZMP in the magnetic field. With the obtained DNA-linked magnetic nanoprobe, the labeled amount of enzyme-linked antibodies and detection sensitivity are further enhanced. Antibody leakage can be prevented, and the activity of the antibody can be maintained; (3) The probe can be used for separation, enrichment, and detection. The surface of the immunosensor can be updated after detection by removing matter on the electrode surface via the external magnetic field. The described method was found to be useful for detection with good fabrication reproducibility. It is fast and simple and can be further developed for practical clinical detection of serum tumor marker levels.

## Figures and Tables

**Table 1:** Comparison of the current one-dimensional magnetic electrochemical immunosensor to other sandwich-type immunosensors for CEA detection.

Immunosensor fabrication	Linear range (ng/mL)	DL (ng/mL)	ref.
HRP-anti-CEA/nano-Au/CS/CPCE	0.5–25	0.22	[19]
anti-CEA/protein A/nano Au/ carbon fiber microelectrode (CFME)	0.01–160	0.005	[20]
HRP–anti-CEA/nanogold/chitosan/SPCE	2.5–40.0	1.1	[21]
anti-CEA/double nano-Au/poly(diallyldimethylammonium chloride /GCE	0.5–120	0.2	[22]
HRP-anti-CEA/GA/CoFe_2_O_4_/CPE	1.5–60	0.5	[23]
anti-CEA/Au/Fe_3_O_4_ NPs/SPCE	0.01–150	0.005	[24]
anti-CEA/nano-Au/NiHCF/nano-Au/GCE	0.5–160	0.1	[25]
This method	0.008–200	0.005	Our work

**Table 2:** Results of CEA detection in human serum with our ECI compared with ELISA (*n* = 3).

	CEA concentration (ng/mL)				
	
Sample	Detected by this method	Detected by ELISA	[CEA] Added in serum	Found after add CEA	Recovery (%) a
1	2.1	2.0	2.0	4.3	105
2	4.2	4.0	4.0	8.0	95
3	10.2	10.5	10.0	21	107

a Recovery = 100% × (found − this method)/added.
